# Syphilis in Ancient and Prehistoric Times

**Published:** 1892-09

**Authors:** 


### BOOK NOTICES.

SYPHILIS IN ANCIENT AND PRE-HISTORIC TIMES. By Dr. F. Buret. Translated from the French, with notes by A. H. Ohmann-Dumesnil, M. D. In three volumes. Volume I. *Syphilis To-day and Among the Ancients* (No. 12 in the Physicians' and Students' Ready Reference Series). Philadelphia (1231 Filbert Street) and London: F. A. Davis, Publisher. 1891. Price, cloth, $1.25 net.

This first volume of the new work on that ever-interesting medical subject, "Syphilis," is sufficiently characterized by its title. It is a most able attempt to show that all our notions about syphilis having been introduced among civilized men at any given modern period of the world's history as commonly taught by different investigators are entirely wrong. On the contrary syphilis is as old as the human race.

The author gives exhaustive researches to show that it was known to the most ancient peoples, or rather that its ravages were known to them, though they did not know enough to group all the various manifestations which it gave in different individuals under one name.

He quotes from the oldest poets most significant, though obscure, sentences, referring in humorous or sarcastic terms to the pathological afflictions of different individuals and with pointed hints at the disgraceful origin of these afflictions. To the trained medical eye these allusions can mean but one disease—syphilis. He quotes certain passages of Holy Writ in which the same obscure allusions occur and are susceptible of the same explanation.

He even examines the bones of pre-historic man which have from time to time been discovered and finds upon them the furrows and notches of syphilitic ulcerations, and thus comes to the inevitable conclusion that syphilis is old as man.

No one who reads the book can lay it aside without having received the conviction that the author has arrived at the truth of the question.

The book is cleverly written. All through its pages may be detected much of the humorous style of another one of the excellent members of this series—"Circumcision," by Remondino—before reviewed in the pages of this journal.

It is printed and bound uniform with the rest of the series, and is altogether an admirable publication. W. M. J.

